# Evaluation of ^161^Tb-Labeled Diphosphonates as Potential Bone-Targeting Agents

**DOI:** 10.3390/ijms262110392

**Published:** 2025-10-25

**Authors:** Pavle Sitarica, Aleksandar Vukadinović, Miloš Marić, Sanja Vranješ-Đurić, Dalibor Stanković, Marko Perić, Drina Janković, Dragana Stanković, Marija Mirković, Magdalena Radović

**Affiliations:** 1 “VINČA” Institute of Nuclear Sciences, National Institute of the Republic of Serbia, University of Belgrade, 11001 Belgrade, Serbia; pavle.sitarica@vin.bg.ac.rs (P.S.); vukadinovic@vin.bg.ac.rs (A.V.); milos.maric@vin.bg.ac.rs (M.M.); sanjav@vin.bg.ac.rs (S.V.-Đ.); daliborstankovic@vin.bg.ac.rs (D.S.); markoperic1983@gmail.com (M.P.); drinaj@vin.bg.ac.rs (D.J.); dragana.s@vin.bg.ac.rs (D.S.); mmarija@vin.bg.ac.rs (M.M.); 2 Faculty of Chemistry, University of Belgrade, 11001 Belgrade, Serbia

**Keywords:** HEDP, ZOL, ^161^Tb-labeled diphosphonates, hydroxyapatite binding, biodistribution, bone-targeting, electrochemical behavior, DFT

## Abstract

Two diphosphonates, etidronic acid (HEDP) and zoledronic acid (ZOL), were radiolabelled with ^161^Tb and evaluated as potential bone-targeting radiopharmaceuticals. Radiolabeling was performed at pH 7, achieving high radiolabeling yields (greater than 98%) and demonstrating excellent in vitro stability in saline and human serum. Both radiolabeled complexes exhibited hydrophilic behavior, a strong binding affinity to hydroxyapatite, and moderate to high plasma protein binding. Biodistribution studies in healthy Wistar rats demonstrated that ^161^Tb-HEDP and ^161^Tb-ZOL achieve high and stable skeletal uptake with rapid blood clearance and minimal soft tissue accumulation. ^161^Tb-HEDP favored higher initial bone localization, while ^161^Tb-ZOL showed lower renal and hepatic accumulation, indicating higher safety and selectivity. Compared to unchelated ^161^TbCl_3_, both diphosphonate complexes exhibited significantly higher bone-to-kidney and bone-to-liver ratios, resulting in superior targeting. Complementary experiments with non-radioactive terbium were performed to investigate the redox behavior and confirm complex formation, providing valuable insight into the stability and binding modes of the ligands. Both terbium and the ligands displayed well-defined redox behavior within the potential range of −1 to 1.7 V, with complex formation evidenced by shifts in the oxidation peaks. Density functional theory (DFT) calculations further supported these findings, showing that both phosphonate groups of a ligand coordinate to Tb^3+^, while the hydroxyl groups in HEDP enable intermolecular hydrogen bonding, contributing to additional structural stabilization. Results encourage further investigations of ^161^Tb-labeled diphosphonates as promising candidates for radionuclide therapy of bone metastases and other skeletal diseases.

## 1. Introduction

Bone metastases are common in patients with advanced malignancies, especially prostate, breast, and lung cancers. The illness incurs substantial suffering, such as severe bone pain, skeletal fractures, hypercalcemia, and even the rare but devastating spinal cord compression [1]. Among the available treatment modalities, targeted radionuclide therapy (TRT) has become a valuable option for palliation of bone pain and potentially for the reduction in skeletal tumor burden [2,3,4].

Current radionuclide therapy includes bone-seeking radiopharmaceuticals such as ^153^Sm-ethylenediamine tetramethylene phosphonate (^153^Sm-EDTMP), ^89^Sr, ^186^Re-hydroxyethylidene diphosphonic acid (^186^Re-HEDP) [5], and ^177^Lu-DOTA-zoledronate [6,7]. Guerra et al. have emphasized the role of ^223^Ra and ^177^Lu for the palliative treatment of bone metastases over several examined radionuclides, including the currently used ^89^Sr and ^153^Sm [8]. The advantage of ^177^Lu as the therapeutic radionuclide is its accompanying gamma emission, which allows the visualization of the biodistribution of the therapeutic agent and monitoring the effect of therapy [9]. Another important benefit is its minimal bone marrow suppression during accumulation in skeletal lesions, which is particularly when marrow reserves are limited [10]. In recent years, ^177^Lu-EDTMP has been proposed as a good bone-seeking radiopharmaceutical [11,12]. EDTMP, as a multidentate ligand, possesses the ability to form metal chelates, especially with lanthanides [13,14,15], but it was noticed that in vivo instability of the formed complexes [13,16]. Some phosphonates, i.e., clodronate, pamidronate, and zolendronate, are in use in non-radiolabeled form in cancer treatment, mainly to reduce elevated blood calcium levels, to strengthen the bone, and to reduce the fracture risk caused by bone metastases. However, very contradictory literature data exist about their effectiveness in bone pain palliation [17,18]. For many years, ^99m^Tc-labeled phosphonates, such as ^99m^Tc-MDP, ^99m^Tc-DPD, ^99m^Tc-HEDP, and ^99m^Tc-EDTMP, have been used in nuclear medicine imaging for the detection and evaluation of metastatic bone cancer. Due to their high sensitivity, bone metastases can be detected before the occurrence of anatomical changes [19,20,21].

Direct radiolabeling of HEDP and ZOL with ^177^Lu (without additional chelators) has been investigated to improve skeletal targeting due to their high binding affinity for hydroxyapatite, making them effective ligands for the relief of bone pain [22,23,24].

To the best of our knowledge, no data are currently available regarding the labeling of bisphosphonates with ^161^Tb. ^161^Tb is quite similar to ^177^Lu in several aspects: it has a comparable half-life (^177^Lu: 6.64 days; ^161^Tb: 6.95 days), mean beta-radiation energy (^177^Lu: 134 keV; ^161^Tb: 154 keV), and maximum specific activities that could be achieved (^177^Lu: max 4.109 PBq/g; ^161^Tb: max 4.356 PBq/g). The ^161^Tb advantage lies in the high number of very low-energy (mostly ≤ 40 keV) conversion and Auger electrons (average about 10 Auger electrons per decay) with high linear energy transfer, extending up to about 30 μm from the decay site, which could contribute to its high therapeutic effectiveness through localized energy deposition [25,26,27,28]. The most relevant γ-lines for SPECT imaging are observed at 48.9 keV, 57.2 keV, and 74.6 keV [29]. By adding ^161^Tb to the current repertoire of radionuclides for palliative treatment of metastatic bone disease, this approach broadens the spectrum and availability of therapies for patients and represents a promising strategy to improve palliative care in metastatic bone disease. This research could pave the way for more effective treatments for cancer patients who currently have no viable options with standard therapies.

Additionally, the lanthanides are chemically very similar, and analogous to Lu^3+^ ions, Tb^3+^ ions are also oxygen seekers and phosphate groups (containing oxygen) of HEDP and ZOL are available for coordination with Tb^3+^. Therefore, we assume that both HEDP and ZOL can be directly labeled with ^161^Tb without the need for additional chelators. This approach allows simple radiolabeling procedures while preserving the intrinsic high bone affinity of these bisphosphonates.

The aim of this study is therefore to investigate the radiolabeling, stability, and in vivo biodistribution of ^161^Tb-HEDP and ^161^Tb-ZOL, supported by complementary experiments with non-radioactive terbium to confirm complex formation. By combining the high skeletal targeting affinity of bisphosphonates with the favorable radiotherapeutic properties of ^161^Tb, their stable complex may represent a promising strategy for treating skeletal micrometastases and contribute to current approaches for bone pain palliation.

## 2. Results

### 2.1. Cyclic Voltammetry Measurements Results

**Figure 1:**
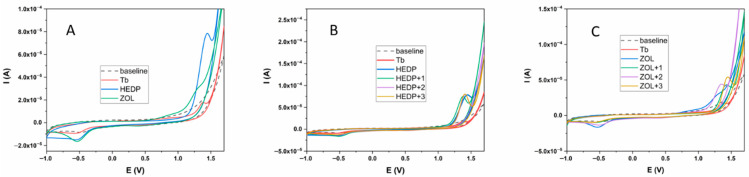
Electrochemical behavior of Tb, HEDP, and ZOL ligands (**A**); HEDP ligand in the presence of Tb (**B**); ZOL ligand in the presence of Tb (**C**). Glassy carbon served as the working electrode, with a carbonate buffer (pH 9) as the supporting electrolyte.

All three tested compounds (Tb, HEDP, and ZOL) showed an electrochemical response in the potential range from −1 to +1.7 V (Figure 1A). In the positive potential region, Tb showed a clearly defined oxidation peak at a potential value of 1.39 V, while the HEDP ligand also showed an oval-shaped, clearly defined peak at a potential of 1.45 V. The ZOL ligand also showed oxidation properties, but unlike the previous two compounds tested, two oxidation peaks were observed, at potentials of 0.93 and 1.27 V. In addition, these peaks were not as clearly defined as in the previous two cases. In the opposite direction, all three tested compounds showed an electrochemical response. In the case of Tb and HEDP ligands, reduction peaks were observed at potentials of −0.56 and −0.54 V, respectively. However, unlike the oxidation direction, these peaks were not as clearly defined. On the other hand, the ZOL ligand had two reduction peaks at potentials of 0.41 V and −0.54 V. The second of these peaks was clearly defined and oval-shaped. The values of the oxidation-reduction potentials are given in Table 1. The formation of Tb complexes with HEDP and ZOL ligands was examined under the same experimental conditions. The results are shown in Figure 1B,C. The experiment with both ligands was performed by successively adding 1 equivalent of Tb of the same concentration to a 1 mM solution of the ligand.

### 2.2. Radiolabeling Yield

Labeling of HEDP and ZOL with ^161^Tb was performed at pH 7. The radiolabeling yield exceeded 98% for both compounds, with ^161^Tb-HEDP achieving 98.64 ± 0.71% and ^161^Tb-ZOL 99.13 ± 1.12% (*n* = 3). Due to the successful radiolabeling and minimal presence of free ^161^Tb^3+^, no additional purification was required.

### 2.3. Radioelectrophoresis Profiles

**Figure 2:**
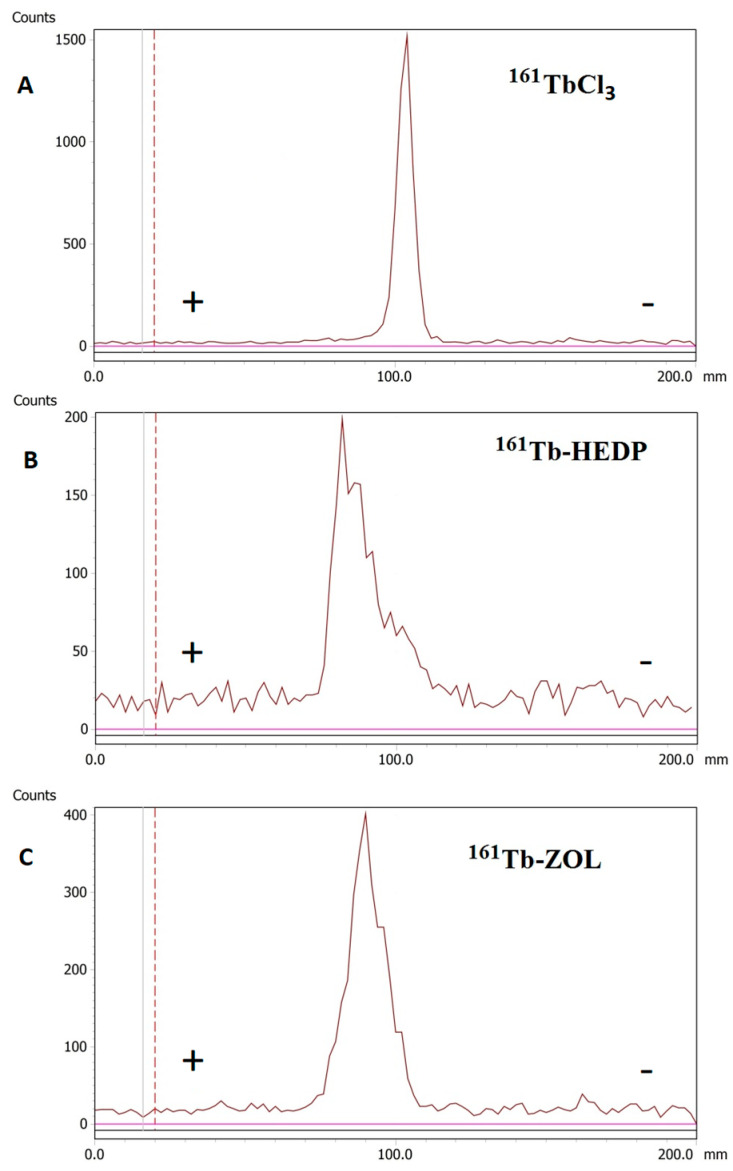
Radioelectrophoresis: (**A**) ^161^TbCl_3_, (**B**) ^161^Tb-HEDP, and (**C**) ^161^Tb-ZOL.

Radioelectrophoresis demonstrated that both ^161^Tb-HEDP and ^161^Tb-ZOL migrated predominantly toward the anode, confirming their net negative charge at pH 7.4. In contrast, free ^161^TbCl_3_ remained at the point of application (labeled as 100.0 in Figure 2) under identical conditions, indicating the formation of electrically neutral hydrolyzed species.

### 2.4. Protein Binding and Lipophilicity

The plasma protein binding values for ^161^Tb-HEDP (75.69 ± 1.51%) and ^161^Tb-ZOL (59.13 ± 1.84%) indicate moderate to high affinity toward plasma proteins. Log *p* values indicated the highly hydrophilic nature of both complexes, with values of −3.16 ± 0.08 for ^161^Tb-HEDP and −3.22 ± 0.06 for ^161^Tb-ZOL. This suggests that they are preferentially cleared by the kidneys and have limited ability to cross lipid membranes [30].

### 2.5. Hydroxyapatite Binding

**Figure 3:**
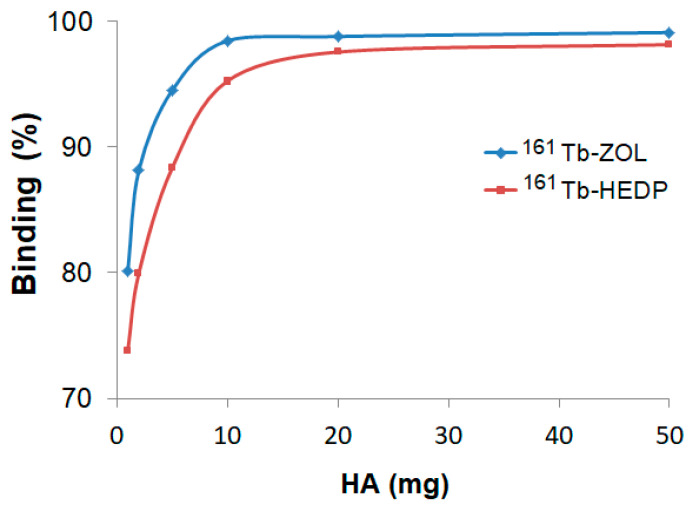
Hydroxyapatite binding of ^161^Tb-diphosphonate complexes after 24 h incubation at different HA amounts.

Hydroxyapatite (HA) binding studies confirmed the strong bone-targeting affinity of both ^161^Tb-HEDP and ^161^Tb-ZOL complexes (Figure 3). However, distinct differences in their binding kinetics and saturation behavior were observed. In vitro binding, assessed after 24 h using increasing amounts of HA (1–50 mg), showed a clear dose-dependent increase for both complexes.

^161^Tb-ZOL shows significantly higher binding efficiency at lower HA concentrations, achieving over 94% binding with just 5 mg of HA and reaching a plateau of ~99% at 10 mg and above. In contrast, ^161^Tb-HEDP shows slightly lower binding percentages at lower HA amounts, reaching 88% at 5 mg and approaching 98% at 50 mg. Although it showed high affinity at elevated HA levels, the slower attainment of maximum binding indicates a relatively lower initial affinity compared to ^161^Tb-ZOL.

### 2.6. In Vitro Stability

The stability of ^161^Tb-HEDP and ^161^Tb-ZOL was evaluated in saline and human serum at 37 °C and room temperature over 96 h. Both complexes maintained >96% radiochemical purity at room temperature and after 24 h incubation in saline. A gradual decrease in radiochemical purity was observed in serum, with values of 94.85 ± 0.95% for ^161^Tb-HEDP and 96.55 ± 0.88% for ^161^Tb-ZOL after 96 h, suggesting in vitro stability under physiological conditions (Table 2).

### 2.7. Biodistribution Profiles

The in vivo biodistribution of ^161^Tb-labeled complexes (^161^Tb-ZOL and ^161^Tb-HEDP at pH 7.0) and free ^161^TbCl_3_ (at pH 7.5 and pH 4.5) was evaluated in healthy Wistar rats at various post-injection time points (2 h, 24 h, and 7 d) following intravenous administration.

**Figure 4:**
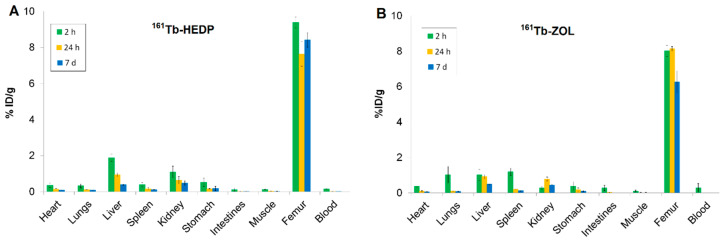
Biodistribution of (**A**) ^161^Tb-HEDP and (**B**) ^161^Tb-ZOL at pH 7 in healthy Wistar rats at 2 h, 24 h, and 7 d. Data are expressed as mean ± SD, *n* = 3 animals per group. %ID/g = percentage of injected dose per gram of tissue.

High skeletal uptake of ^161^Tb-HEDP (Figure 4A) was observed as early as 2 h post-injection (9.39 ± 0.30 %ID/g), with significant retention at 24 h and 7 d (ranging from 7.64 ± 0.68 to 8.42 ± 0.40 %ID/g), indicating strong and stable binding to bone mineral. Blood clearance was rapid and efficient, with activity decreasing from 0.16 ± 0.01 %ID/g at 2 h to nearly undetectable levels by 24 h and remaining negligible at 7 d. This fast systemic clearance is advantageous, as it reduces both non-target radiation exposure and systemic toxicity. Liver uptake (1.89 ± 0.20 %ID/g) and kidney uptake (1.09 ± 0.33 %ID/g) observed at 2 h post-injection suggest involvement of both renal and hepatic excretion pathways. The activity in these organs decreased significantly over time, indicating effective clearance. Other soft tissues, including the heart, lungs, spleen, gastrointestinal tract, and muscle, exhibited minimal uptake throughout the study, underscoring the high selectivity of ^161^Tb-HEDP for bone tissue.

The highest bone accumulation of ^161^Tb-ZOL (Figure 4B) was observed at 24 h post-injection (8.13 ± 0.13 %ID/g), while stable retention was maintained even after 7 d (6.26 ± 0.63 %ID/g), indicating strong and prolonged localization in bone. Blood clearance was rapid, with activity decreasing from 0.29 ± 0.14 %ID/g at 2 h to nearly undetectable levels at 7 d, reflecting efficient systemic elimination and minimal residual circulation. Uptake in the kidneys, liver, and spleen at 2 h (0.28 ± 0.09 %ID/g, 1.03 ± 0.31 %ID/g, 1.19 ± 0.21 %ID/g, respectively), reflects dual renal and non-specific RES clearance with a decrease over time. Minimal accumulation was detected in the heart and gastrointestinal tract, indicating low non-target distribution.

**Figure 5:**
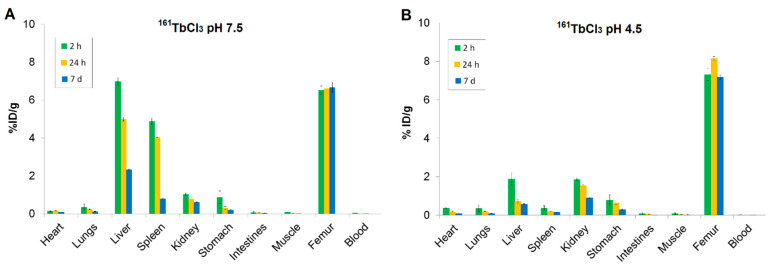
Biodistribution of (**A**) ^161^TbCl_3_ at pH 7.5 and (**B**) ^161^TbCl_3_ at pH 4.5 in healthy Wistar rats at 2 h, 24 h, and 7 d. Data are expressed as mean ± SD, *n* = 3 animals per group. %ID/g = percentage of injected dose per gram of tissue.

Biodistribution studies of ^161^TbCl_3_ at both physiological (pH 7.5) and slightly acidic (pH 4.5) conditions (Figure 5) revealed high and stable bone uptake at all time points (2 h, 24 h, 7 d), confirming strong affinity for bone tissue.

At pH 7.5, the bone uptake of ^161^TbCl_3_ remained high at all time points (6.53 ± 0.22 %ID/g at 2 h; 6.61 ± 0.14 %ID/g at 24 h; 6.68 ± 0.29 %ID/g at 7 d), while blood activity levels were consistently low, indicating rapid systemic clearance. Liver and spleen showed high initial uptake (6.99 ± 0.21 and 4.88 ± 0.15 %ID/g at 2 h, respectively), followed by a gradual decrease (liver: 2.33 ± 0.02 %ID/g; spleen: 0.79 ± 0.02 %ID/g at 7 d), indicating that both organs are involved in early-phase uptake. The early uptake in the kidneys, which decreased over time (1.03 ± 0.06 %ID/g at 2 h and 0.60 ± 0.02 %ID/g at 7 d), indicates a renal excretion route. Other tissues, including lungs, heart, gastrointestinal tract, and muscle, showed low to negligible retention, indicating minimal nonspecific accumulation (Figure 5A).

At pH 4.5, bone uptake of ^161^TbCl_3_ was slightly higher (7.31 ± 0.32 %ID/g at 2 h) and retention in bone was sustained for 7 d (7.18 ± 0.12 %ID/g). In contrast, blood shows very low %ID/g values across all time points, indicating rapid clearance from systemic circulation. The liver and spleen showed initial lower accumulation compared to pH 7.5 (1.88 ± 0.31 %ID/g and 0.37 ± 0.12 %ID/g, respectively), followed by a significant decrease. The kidneys uptake at 2 h (1.85 ± 0.07 %ID/g), with a gradual decrease over time, indicates renal excretion as the predominant clearance route. Uptake in other tissues remained low (Figure 5B).

Bone-to-organ ratios offer a more accurate indication of targeting efficacy and selectivity (Table 3). Among the tested compounds, ^161^Tb-ZOL demonstrated the highest bone-to-kidney and bone-to-liver ratios (28.64 and 7.79, respectively), which are crucial for assessing safety and minimizing non-target radiation. These values, which were significantly higher than those of ^161^TbCl_3_ (pH 4.5) (3.97 and 3.89) and ^161^Tb-HEDP (8.61 and 4.97), highlight the exceptional selectivity of ^161^Tb-ZOL. The bone-to-muscle ratio remained consistently high (more than 75%) throughout all three formulations, indicating efficient skeletal targeting relative to soft tissue.

### 2.8. Results of DFT Calculations

**Figure 6:**
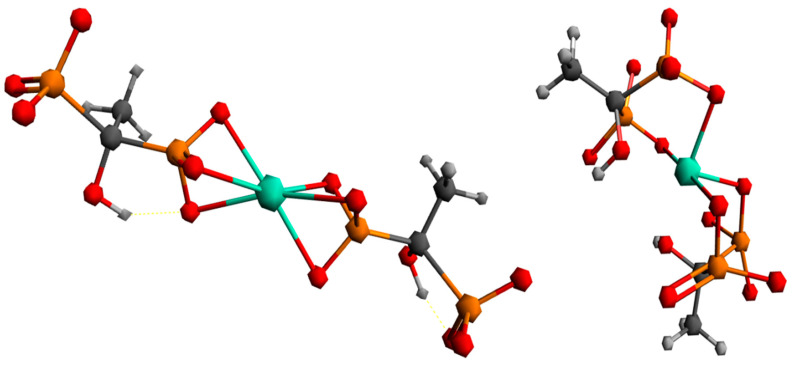
Proposed ways of coordination for the Tb^3+^ complex with the HEDP ligand.

To better understand the coordination chemistry of terbium with phosphonate ligands, two initial structures were analyzed, shown in Figure 6.

**Figure 7:**
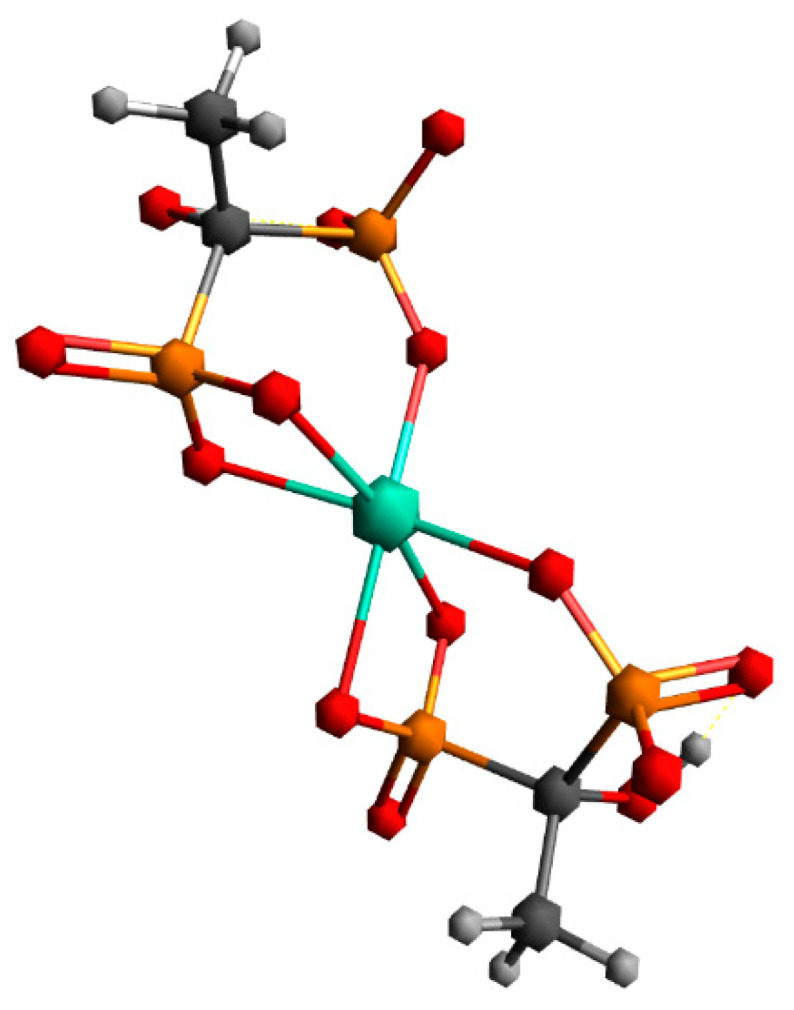
Geometry obtained through energy minimization of the second proposed structure.

The first structure assumed the coordination of two HEDP ligands in a tridentate manner, via three oxygens of one phosphonate group of each HEDP. The second structure assumed octahedral coordination with two HEDP ligands. Each HEDP ligand is coordinated in a tridentate manner, via oxygen atoms from two phosphonate groups of one HEDP and a hydroxyl group from that molecule. The geometric optimization of the first structure did not lead to any changes in the way of coordination. In further text, this complex would be designated with the number 1. The absence of negative vibrational modes indicated that the optimization resulted in an energy minimum. However, during the optimization of the second structure, which included coordination through hydroxyl groups, a complete reorganization occurred, which indicated a completely different way of coordination Figure 7. This way of binding the HEDP ligand is in a tridentate manner via two oxygens of one phosphonate group and one oxygen of another phosphonate group of the same molecule. In further text, this complex would be designated with the number 2.

Comparing the first and second obtained structures, it could be seen that the first structure has about 55 kcal/mol more energy than the second one.

Based on the data given in Table 4, it is evident that nuclear repulsions favor structure 1, while electronic stabilization favors structure 2. However, entropic factors favor structure 1, with the largest contribution being vibrational entropy.

## 3. Discussion

The radiolabeling yields of both ^161^Tb-HEDP and ^161^Tb-ZOL exceeded 98%, even under mild conditions (pH ~7, room temperature), highlighting the strong affinity of ^161^Tb to coordinate with diphosphonate ligands. Maintaining the reaction pH around 7 was essential for achieving high radiolabeling yield, as lower pH levels inhibit deprotonation of the phosphonate groups, while higher pH values increase the risk of lanthanide hydrolysis and colloid formation [31,32]. An excess of diphosphonate ligand during ^161^Tb radiolabeling was used to ensure efficient complex formation and prevent terbium ion hydrolysis. At nearly neutral pH, lanthanides commonly develop insoluble hydroxides, so the presence of excess ligand stabilizes ^161^Tb^3+^ in solution, resulting in high radiochemical yield and improved in vivo stability of the radiocomplex. This aligns with earlier studies involving ^177^Lu, which exhibits similar coordination properties to ^161^Tb, where diphosphonates have been radiolabeled under comparable conditions [22,24].

The absence of unbound ^161^Tb or colloidal impurities was confirmed by ITLC and radioelectrophoresis, underlining the efficiency and repeatability of the direct radiolabeling protocol. Radioelectrophoresis showed that both radiocomplexes exhibited a strong negative charge at pH 7.5, as expected due to the coordination of ^161^Tb^3+^ with phosphonate groups, while free ^161^TbCl_3_ remained stationary, likely as neutral or hydrolyzed species. This distinction is important because it confirms the formation of stable, charged complexes essential for in vivo behavior.

Lipophilicity measurements confirmed the highly hydrophilic nature of both complexes (log P~−3.2), which correlates with their poor membrane permeability and suggests renal clearance. These characteristics are ideal for bone-seeking agents, as they limit non-target distribution and minimize nonspecific tissue accumulation. In vitro protein binding studies revealed moderate to high plasma protein affinity of ^161^Tb-HEDP (75.69 ± 1.51%) and ^161^Tb-ZOL (59.13 ± 1.84%). These values would suggest prolonged circulation time and slower systemic clearance compared to low-binding compounds, potentially enhancing bone accumulation while reducing early renal excretion [33].

The results of the hydroxyapatite (HA) binding studies provide strong evidence for the bone-targeting capabilities of both ^161^Tb-labeled diphosphonates. This strong affinity is attributed to the diphosphonate structure of HEDP and ZOL, which provides phosphonate groups capable of chelating to the calcium ions present in hydroxyapatite crystals [34]. High HA binding efficiency directly correlates with the favorable in vivo biodistribution profile in bone-targeted applications, combining rapid skeleton accumulation with efficient clearance from soft tissues and blood [35]. Results suggest a higher initial affinity of the ^161^Tb-ZOL for bone mineral, particularly at lower HA concentrations. This enhanced interaction is likely due to the presence of additional functional moieties in the zoledronate structure, especially the hydroxyl side chain and nitrogen-containing heterocycle, which can facilitate more robust coordination with calcium ions and contribute to increased electrostatic and hydrogen bonding interactions. These results agree with earlier studies showing that hydroxylated bisphosphonates form more stable lanthanide complexes and exhibit stronger HA binding both in vitro and in vivo [36,37]. Rapid and efficient localization to bone is critical for therapeutic efficacy in conditions such as skeletal metastases, where high specificity and retention in osseous tissue are essential.

Both radiocomplexes displayed excellent in vitro stability in saline and human serum at 37 °C over 96 h. Radiochemical purity remained >94% throughout the study, indicating resistance to transchelation or degradation under physiological conditions. Stability in serum is particularly crucial, as premature decomplexation could result in increased uptake in non-target organs and reduced therapeutic efficacy.

The biodistribution profiles of ^161^Tb-diphosphonate complexes (^161^Tb-ZOL and ^161^Tb-HEDP) and pure ^161^TbCl_3_ (at pH 4.5 and 7.5) reveal important differences in pharmacokinetics, tissue selectivity, and safety that are critical for their potential use in bone-targeted radionuclide therapy.

Both ^161^Tb-ZOL and ^161^Tb-HEDP demonstrated very high and sustained bone uptake (≥6.5 %ID/g) similar to ^177^Lu-EDTMP [11,12], with peak accumulation occurring between 2 and 24 h post-injection and remaining high for up to 7 days. This is attributed to the diphosphonate structure of the ligands (ZOL and HEDP), which contain phosphonate groups capable of strong binding to hydroxyapatite, the principal mineral component of bone [37].

^161^Tb-HEDP showed higher initial bone accumulation, making it suitable for treatments requiring rapid radiation delivery. In contrast, ^161^Tb-ZOL exhibited higher bone-to-kidney ratios, indicating reduced renal burden. Both complexes cleared rapidly from the blood and showed minimal retention in soft tissues (muscle, gastrointestinal tract), which is essential for limiting non-target organ toxicity. While blood protein binding can influence circulation time, the affinity for bone mineral dictates its bone targeting. The noticeable hepatic uptake of ^161^Tb-diphosphonates likely results from partial in vivo dissociation, where released ^161^Tb^3+^ undergoes hydrolysis and is subsequently cleared by the reticuloendothelial system (RES) [38].

At physiological pH (7.5), ^161^TbCl_3_ undergoes rapid hydrolysis, forming insoluble hydroxide and colloidal species, which are readily recognized and cleared by the reticuloendothelial system, mainly the liver and spleen. This non-specific organ uptake increases radiation exposure to healthy tissues and reduces targeting precision. Despite this, ^161^TbCl_3_ also showed high bone uptake due to terbium’s natural affinity for phosphate groups in hydroxyapatite. However, the absence of a stabilizing ligand leads to lower selectivity and greater RES accumulation.

Decreasing the pH to 4.5 improves ^161^TbCl_3_ solubility, preventing the formation of insoluble hydroxides. This results in higher early kidney uptake (peaking around 2 h) and faster urinary excretion of soluble terbium species. Liver and spleen uptakes remain present but at reduced levels compared to pH 7.5, indicating less hydrolysis. However, even at acidic pH, ^161^TbCl_3_ lacks targeting specificity and demonstrates higher soft tissue distribution compared to the diphosphonate complexes, limiting its suitability for clinical use without appropriate chelation. Strong, kinetically stable chelators like diphosphonates enable precise delivery of therapeutic radionuclides while minimizing systemic toxicity, making them the preferred choice for clinical development.

Bone-to-organ ratios provide a quantitative measure of radiopharmaceutical selectivity and safety. A high bone-to-kidney ratio reflects strong skeletal uptake with minimal renal retention, reducing the risk of nephrotoxicity. Similarly, a high bone-to-liver ratio indicates stable, bone-specific accumulation with limited hepatic sequestration, minimizing RES involvement and potential hepatotoxicity. Together, these ratios highlight the compound’s ability to deliver radiation efficiently to bone while sparing critical non-target organs. Comparison of the biodistribution profiles of ^161^Tb-HEDP, ^161^Tb-ZOL, and free ^161^TbCl_3_ at pH 4.5 reveals clear differences in skeletal selectivity, clearance pathways, and non-target organ retention. Although ^161^Tb-HEDP showed the highest initial bone accumulation, this advantage was offset by moderate uptake in both kidneys and liver, which may limit its therapeutic safety margin. Free ^161^TbCl_3_ also localized in bone, but exhibited pronounced accumulation in excretory organs, reflected in its lowest bone-to-kidney (3.97) and bone-to-liver (3.89) ratios, underscoring its limited selectivity and in vivo stability. In contrast, ^161^Tb-ZOL provided the most favorable overall profile: despite slightly lower bone uptake compared to HEDP, it demonstrated significantly lower renal and hepatic retention, resulting in the highest bone-to-kidney (28.64) and bone-to-liver (7.79) ratios.

Taken together, these findings highlight ^161^Tb-ZOL as the most promising candidate for bone-targeted radionuclide therapy, offering an optimal balance of skeletal accumulation, complex stability, and low non-target organ retention compared to both ^161^Tb-HEDP and free ^161^TbCl_3_ (pH 4.5). In addition to this favorable pharmacokinetic behavior, ^161^Tb provides a unique dosimetric profile that combines the advantages of both α^−^ and β^−^ emitters for bone-targeted therapy. Unlike ^223^Ra, which delivers highly cytotoxic α-particles with very short ranges (~50–100 µm) primarily to osteoclasts at the bone surface but limited cross-fire to adjacent tumor cells, ^161^Tb provides enhanced microdosimetric coverage of tumor cells near the bone surface via low-energy Auger and conversion electrons, in addition to β^−^ emissions. Compared to ^153^Sm, which has a longer β^−^ range (~0.8 mm) and allows some cross-fire but with lower energy deposition, ^161^Tb achieves higher local absorbed doses at the micrometer scale (20–40% increase) while maintaining limited penetration into deeper marrow. From a dosimetric perspective, this energy deposition effectively irradiates tumor cells adherent to mineralized bone while also delivering a therapeutic dose to osteogenic cells involved in bone remodeling, such as osteoclasts and osteoblasts. This combination ensures a steep dose gradient, maximizing tumoricidal effects at the bone-tumor interface while sparing healthy marrow and soft tissue, positioning ^161^Tb-phosphonates as a promising next-generation agent for palliative and potentially therapeutic management of bone metastases [25,26].

In addition, the electrochemistry study of the synthesized complexes provided essential insights into the redox behavior of non-radioactive terbium and its interaction with phosphonate ligands. The observed and well-defined oxidation/reduction peaks, along with their potential shifts upon complexation, confirmed the successful formation of stable terbium–diphosphonate complexes. The obtained values for the electrochemical behavior of the tested compounds are in good agreement with the values given so far for these compounds [39,40,41,42]. As with the HEDP ligand, the first equivalent of Tb shows the formation of the complex causing a shift in the oxidation peak to lower potential values of +1.37 V. Further addition of Tb does not cause a shift in the potential to a value of +1.39 V, which is the same value as for Tb alone, implying that the obtained peak is the resulting peak of the superposition of the oxidation peak of the formed complex and Tb. Further addition of Tb does not cause any changes in the electrochemical behavior of the system. From the obtained results, it can be concluded that the complex is formed in the ratio 1/1, which is in accordance with the results obtained for the Eu/HEDP system under basic conditions [43]. Additionally, no or very small changes were observed in the reduction behavior, which suggests that the formation of the complex can be monitored via the oxidation behavior of the metal and ligand. A different behavior was obtained for the Tb/ZOL system. After the addition of the first equivalent of Tb, the formation of the complex is clearly visible by obtaining a new oxidation peak at a potential value of +1.42 V, without affecting the reduction potential of ZOL. The second equivalent of Tb causes an additional shift in the oxidation peak to lower potential values of +1.34 V and a clearly defined reduction peak at a value of −0.50 V. The absence of the first reduction peak in both cases is clearly noticeable. The third equivalent of Tb causes a shift in the oxidation peak to +1.40 V and an increase in the current of the second reduction peak, which forms the electrochemical behavior of the system similar to the case of the Tb/HEDP complex. From these results, it can be assumed that two equivalents of Tb participate in the formation of the Tb/ZOL complex, as is the case for the Zn/HEDP system [44]. However, for clearer statements, the literature data for similar systems with the ZOL ligand, as well as additional research, are lacking.

**Figure 8:**
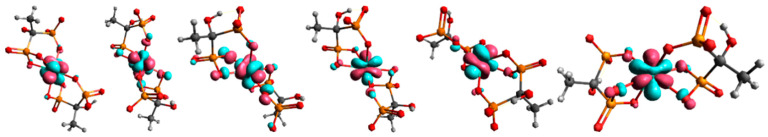
Molecular orbitals with dominant f-character for complex 2. Only the orbitals containing one unpaired electron are depicted in the figure, since the Tb^3+^ ion has an f^8^ configuration.

Building upon these findings, the DFT analysis was employed to gain a deeper molecular-level understanding of the binding modes and stabilization factors underlying complex formation. In structure 1, the non-coordinated phosphonate groups are oriented in such a way that they are maximally distant from terbium and other phosphonate groups. By moving to structure 2, all phosphonate groups are brought closer together, which can lead to an increase in nuclear repulsive interactions. At the same time, the binding of all phosphonate groups to terbium in structure 2 leads to a decrease in electronic energy. In the case of uncoordinated phosphonate groups, electronic repulsive interactions are greater due to the negative charge on the oxygen atoms. By coordination to the metal, the negative charge on the oxygen atoms is partially neutralized, which reduces repulsive electronic interactions. Furthermore, the transition from a four-membered ring, which is under large tension, to a six-membered ring in structure 2 causes a lowering of electronic repulsive interactions. The vibrational entropy of a ligand typically decreases when it binds to a metal. This reduction in entropy is due to the ligand becoming more constrained in its movements upon forming a bond with the metal, limiting the number of vibrational modes available. Vibrational entropy is related to the number and types of vibrations a molecule can undergo. These vibrations include stretching, bending, and twisting of bonds. When a ligand binds to a metal, it forms a complex where the ligand is held in a specific orientation and conformation. This binding restricts the ligand’s ability to freely rotate, translate, and vibrate, leading to a decrease in the number of accessible vibrational modes. This is exactly what happens when free phosphonate groups coordinate to terbium. Our findings and geometry analysis are in accordance with available literature data [45,46]. The dominant interaction in lanthanide-ligand bonding is electrostatic, meaning it is primarily due to the attraction between the positively charged lanthanide ion and the negatively charged or electron-rich portions of the ligand. Lanthanides act as hard Lewis acids, favoring interactions with hard ligands (those with high charge density and low polarizability). The 4f electrons in lanthanides are deeply buried within the atom’s core and are generally not involved in bonding interactions Figure 8.

The ionic radius of the lanthanide ion plays a significant role in binding with bisphosphonate-type chelating ligands. Due to lanthanide contraction, ΔG for the interchange between structures 1 and 2 can significantly vary through the lanthanide series. Thus, for example, the ΔG for the transition from structure 1 to structure 2 for equivalent Lu^3+^ complexes is ≈−61 kcal/mol (DFT analysis at the same level of theory). These results may indicate possible differences in the labeling of bisphosphonate ligands with ^161^Tb^3+^ and ^177^Lu^3+^.

While electrochemistry revealed macroscopic redox behavior, DFT calculations enabled visualization of ligand–metal interactions, confirming the preferential coordination of phosphonate groups to Tb^3+^ and highlighting the stabilizing role of hydroxyl groups through hydrogen bonding.

## 4. Materials and Methods

### 4.1. Reagents and Instruments

1-Hydroxyethylidene-1,1-diphosphonic acid (etidronic acid, HEDP), 1-hydroxy-2-(1H-imidazol-1-yl)ethane-1,1-diyldiphosphonic acid (zoledronic acid, ZOL), and terbium(III) chloride hexahydrate (TbCl_3_·6H_2_O) were purchased from Sigma-Aldrich (St. Louis, MO, USA) and used without further purification. No-carrier-added ^161^TbCl_3_ solution in 0.05 M HCl (20.66 GBq/mL) was provided by Terthera, Breda, The Netherlands. All other reagents and solvents were of analytical grade and used as received. Ultrapure water was obtained from a Milli-Q purification system (Millipore, Billerica, MA, USA).

Radioactivity of the samples was measured using a dose calibrator (CRC-15R beta, Capintec Inc., Ramsey, NY, USA). A gamma counter (WIZARD 2480, PerkinElmer, Shelton, CT, USA) was used for biodistribution studies and quantification of radioactivity in aliquots. Radio-thin layer chromatography (radio-TLC) of ^161^TbCl_3_ and ^161^Tb-diphosphonate complexes was performed using a Scan-RAM radio-TLC scanner (LabLogic Group, South Yorkshire, UK).

### 4.2. Evaluation of Complex Formation by Cyclic Voltammetry

The electrochemical behavior of Tb, as well as ZOL and HEDP ligands, was investigated using a three-electrode system, comprising a glassy carbon working electrode, an Ag/AgCl (3 M KCl) reference electrode, and a platinum wire counter electrode. A carbonate buffer at pH 9 serves as the supporting electrolyte. Before each measurement, the glassy carbon electrode was carefully polished with 0.3 μm and 0.05 μm aluminum oxide paste, followed by ultrasonic cleaning and thorough rinsing with distilled water.

### 4.3. Radiolabeling of Diphosphonates

For the labeling of diphosphonates (HEDP and ZOL) with ^161^Tb, each phosphonate (5 mg) was first dissolved in 0.5 mL of sodium carbonate-bicarbonate buffer (pH 9). The pH was then adjusted to 7 using 0.1 M HCl. Radiolabeling reactions were performed by mixing ^161^TbCl_3_ solution containing the required activity (typically 7.4–74 MBq, corresponding to 200 μCi–2 mCi) with diphosphonate ligand and incubating at room temperature (22–24 °C) for 30 min with gentle mixing.

### 4.4. Characterization of ^161^Tb-Diphosphonate Complexes

#### 4.4.1. Assessment of Radiochemical Purity by Instant Thin-Layer Chromatography (ITLC)

Radiochemical purity of the radiolabeled complexes was assessed using instant thin-layer chromatography (ITLC-SG, Agilent Technologies, Santa Clara, CA, USA). The analysis was conducted on silica gel (SG) strips (2 × 14 cm) using ammonia/ethanol/water (1:10:20, *v*/*v*/*v*) as the mobile phase to separate free ^161^Tb^3+^ from ^161^Tb-diphosphonate complexes. For each sample, 5 μL of the radiolabeled solution and a control sample of pure ^161^TbCl_3_ were spotted on the SG strip and developed up to 12 cm. Under these conditions, the ^161^Tb-diphosphonate complexes migrate with the solvent front (Rf ≈ 1.0), while free ^161^TbCl_3_ remains at the origin (Rf ≈ 0.0). After development, the strips were dried and scanned using a TLC scanner.

#### 4.4.2. Determination of Complex Charge by Radioelectrophoresis

Radioelectrophoresis was employed to determine the net charge of the ^161^Tb-labeled diphosphonate complexes and to confirm successful radiolabeling. For each sample, 5 μL of the radiolabeled complex or a control solution of ^161^TbCl_3_ was applied to the center of Whatman 3MM chromatography paper strips (2 × 25 cm), which had been pre-soaked in 0.05 M phosphate buffer (pH 7.4). The strips were placed in an electrophoresis chamber filled with the same phosphate buffer, and electrophoresis was carried out at 250 V for 1 h using a Gelman Instrument electrophoresis system (Gelman Instrument Co., Ann Arbor, MI, USA). After radioelectrophoresis, each strip was dried and scanned using a TLC scanner. This method allowed the determination of the migration pattern of the radiolabeled species compared to free ^161^Tb^3+^, providing information about the charge and integrity of the formed complexes [22].

#### 4.4.3. Determination of Lipophilicity

The lipophilicity (partition coefficient) of the ^161^Tb-diphosphonate complexes was determined by the previously described method [47,48]. A mixture consisting of 0.1 mL of the radiolabeled complex, 0.9 mL of 0.025 M phosphate buffer (pH 7.4), and 1.0 mL of 1-octanol was prepared. The mixture was vortexed for 1 min at room temperature to facilitate the distribution of the radiolabeled compound between the aqueous (buffer) and organic (octanol) phases. Following this, the mixture was centrifuged at 3000 rpm for 5 min to ensure complete phase separation. From both phases, 0.1 mL aliquots were taken, and their radioactivity was measured in a gamma counter. The lipophilicity of the complexes was expressed as the partition coefficient (log P), calculated using the following Formula (1):

log P = log (counts in n-octanol/counts in buffer)(1)

All measurements were performed in triplicate, and the results were reported as mean ± standard deviation.

#### 4.4.4. Protein Binding Determination

Protein binding of the ^161^Tb-diphosphonate complexes was evaluated using the standard TCA precipitation method [47,48]. Aliquots of the radiolabeled complexes (0.1 mL) were added to 0.5 mL of 12% (*w*/*v*) human serum albumin (HSA) solution (provided by the National Blood Transfusion Institute, Belgrade, Serbia) and incubated at 37 °C for 20 min. After incubation, 1 mL of 20% (*w*/*v*) trichloroacetic acid (TCA) was added to precipitate the protein-bound fraction. The mixture was centrifuged for 5 min at 3000 rpm, and the resulting precipitate was rinsed with 0.9% NaCl. This procedure was repeated three times to ensure thorough separation. The radioactivity of both the precipitate-protein-bound fraction (A) and the supernatant-free fraction (B) was measured using a gamma counter. The percentage of HSA-bound ^161^Tb-diphosphonate complexes was calculated using the following Formula (2):

HSA binding (%) = (A/A + B) × 100(2)

#### 4.4.5. Hydroxyapatite Binding Assay

The affinity of ^161^Tb-labeled diphosphonates for bone mineral was tested in vitro using synthetic hydroxyapatite (HA), following a modified version of a previously reported method [49]. Briefly, varying amounts of solid HA (1, 2, 5, 10, 20, and 50 mg) were placed into separate vials. Each vial was then filled with 2 mL of saline solution and shaken for 2 h to equilibrate. After equilibration, 50 µL of the radiolabeled ^161^Tb-diphosphonate complexes were added to each vial. The mixtures were gently shaken at room temperature for 24 h. The suspensions were centrifuged, and the supernatant liquid was taken from each vial, and the radioactivity was measured with a gamma counter. The percentage of hydroxyapatite-bound radiocomplex was calculated using Formula (3), where A is the mean radioactivity of the supernatant (unbound fraction) and B is the total radioactivity initially added to the vial.

HA binding (%) = [1 − (A/B)] × 100(3)

This assay provides a measure of the bone-targeting potential of each ^161^Tb-diphosphonate complex based on its affinity for hydroxyapatite.

### 4.5. In Vitro Stability Studies of ^161^Tb-Diphosphonate Complexes

The in vitro stability of the radiolabeled complexes, ^161^Tb-HEDP and ^161^Tb-ZOL, was evaluated by radiochromatographic analysis described above at appropriate time points (24 and 96 h). Stability was assessed at room temperature as well as after incubation in saline and human serum at 37 °C. For incubation in saline and serum, 0.1 mL of the radiolabeled complex was added to 0.9 μL of the respective medium. The mixtures were incubated at 37 °C for up to 96 h. At the specified time points, samples were analyzed by radiochromatography to determine the percentage of intact complex and evaluate radiochemical stability [22].

### 4.6. Biodistribution Studies

All animal experiments were conducted using healthy Wistar rats weighing 100–120 g. The rats were obtained from the Military Medical Academy (Belgrade, Serbia). All procedures involving animals were approved by the Ethical Committee of the Vinča Institute of Nuclear Sciences (permission No. 002225501 2025, issued on 20 May 2025) and were performed by the Directive 2010/63/EU of the European Parliament and of the Council of 22 September 2010 [50] on the protection of animals used for scientific purposes, as well as national animal welfare laws.

A total of 0.74 MBq of each ^161^Tb-labeled compound (^161^Tb-HEDP and ^161^Tb-ZOL) and free ^161^TbCl_3_ (at pH 7.5 and pH 4.5) was prepared in a final injection volume of 0.1 mL. Physiological saline was added to adjust the concentration to 10 µg of radiolabeled complex per 0.1 mL. Each formulation was administered intravenously via the lateral tail vein.

Animals (*n* = 3 per time point) were sacrificed at predetermined intervals: 2 h, 24 h, and 7 d post-injection, depending on the study group. Euthanasia was performed by intraperitoneal injection of a ketamine/xylazine overdose. Immediately after sacrifice, major organs and tissues (heart, lungs, liver, spleen, kidneys, stomach, intestines, muscle, and femur) were collected, weighed, and assessed for radioactivity using a gamma counter. The biodistribution of radiolabeled compounds was expressed as the percentage of the injected dose per gram of tissue (%ID/g), calculated by comparing the sample activity to that of the standard representing 100% of the injected dose. All data are presented as mean ± standard deviation (SD).

### 4.7. Computational Details

The geometry optimization and energy minimization have been performed at the BP86 [51] level of theory. For the heavy lanthanide Tb^3+^ ion, the SARC2-DKH-QZVP basis set was used, while the DKH-def2-SVP basis set was applied to all other atoms [52]. Also, SARC/J auxiliary basis set, which is a decontracted def2/J auxiliary set, that is more accurate for relativistic calculations, has been used [52,53,54,55,56].

## 5. Conclusions

In this study, we successfully developed and evaluated two ^161^Tb-labeled diphosphonate complexes, ^161^Tb-HEDP and ^161^Tb-ZOL, as potential bone-targeted radiopharmaceuticals. Radiolabeling was achieved under mild conditions with high yields (~98%) and excellent stability in physiological media. Both complexes are hydrophilic, showing moderate-to-high plasma protein binding and demonstrating strong hydroxyapatite affinity.

Biodistribution studies in healthy Wistar rats confirmed their high and stable skeletal accumulation, rapid blood clearance, and minimal non-target organ accumulation. ^161^Tb-HEDP showed higher initial bone uptake and an excellent bone-to-blood ratio, while ^161^Tb-ZOL demonstrated lower renal and hepatic accumulation, offering greater safety and selectivity for bone-targeted therapy. In contrast to unchelated ^161^TbCl_3_, whose biodistribution is strongly pH-dependent, both diphosphonate complexes displayed superior targeting. The key difference between ^161^Tb-diphosphonate complexes and free ^161^TbCl_3_ at pH 4.5 lies in their bone-to-kidney and bone-to-liver ratios. The diphosphonate complexes achieve higher values, reflecting superior stability and skeletal selectivity, whereas free ^161^TbCl_3_ shows lower ratios due to enhanced renal and hepatic uptake. Overall, ^161^Tb-ZOL emerges as the most promising candidate for bone-targeted radionuclide therapy, combining strong skeletal accumulation with high complex stability and minimal non-target retention, outperforming both ^161^Tb-HEDP and free ^161^TbCl_3_.

Complementary electrochemical and DFT studies confirmed complex formation, revealing phosphonate groups as the primary binding sites and indicating the possibility of binuclear complex formation.

Future studies should focus on therapeutic efficacy in bone metastasis models, comparative evaluation with ^177^Lu analogs, and dosimetry to ensure safe and effective clinical translation. With their excellent stability, bone selectivity, and straightforward preparation, ^161^Tb-labeled diphosphonates represent promising next-generation agents for targeted radionuclide therapy of skeletal metastases, offering not only effective treatment but also an additional palliative option for patients with advanced bone malignancies.

## Figures and Tables

**Table 1:** Redox values for Tb, HEDP, and ZOL.

	First Oxidation Pot. (V)	Second Oxidation Pot. (V)	First Reduction Pot. (V)	Second Reduction Pot. (V)
Tb		1.39	/	−0.56
HEDP		1.45	/	−0.54
ZOL	0.93	1.27	0.41	−0.54

**Table 2:** In vitro stability of ^161^Tb-diphosphonate complexes in different media at RT and 37 °C.

Condition	Time (h)	Radiochemical Purity (%)	
		^161^Tb-HEDP	^161^Tb-ZOL
Room temperature	0	98.64 ± 0.71%	99.13 ± 1.12%
	24	97.11 ± 1.05%	98.74 ± 0.85%
	96	95.89 ± 0.94%	97.53 ± 0.99%
Saline, 37 °C	24	96.78 ± 1.24%	98.17 ± 0.62%
	96	95.42 ± 0.83%	97.25 ± 0.74%
Human serum, 37 °C	24	96.09 ± 0.89%	97.81 ± 0.96%
	96	94.85 ± 0.95%	96.55 ± 0.88%

**Table 3:** Bone targeting efficiency of ^161^Tb-diphosphonate complexes and ^161^TbCl_3_ (pH 4.5) 2 h post-injection in healthy Wistar rats. Data represent mean ± SD, *n* = 3.

Parameter	^161^Tb-HEDP	^161^Tb-ZOL	^161^TbCl_3_ (pH 4.5)
Bone uptake (%ID/g)	9.39 ± 0.30	8.02 ± 0.31	7.31 ± 0.32
Blood (%ID/g)	0.16 ± 0.01	0.29 ± 0.14	0.02 ± 0.01
Muscle (%ID/g)	0.12 ± 0.01	0.11 ± 0.05	0.08 ± 0.05
Kidney (%ID/g)	1.09 ± 0.33	0.28 ± 0.09	1.84 ± 0.07
Liver (%ID/g)	1.89 ± 0.20	1.03 ± 0.31	1.88 ± 0.31
Other tissues	Minimal uptake	Minimal uptake	Minimal uptake
Bone-to-kidney ratio	8.61	28.64	3.97
Bone-to-liver ratio	4.97	7.79	3.89

**Table 4:** Gibbs free enthalpies (kcal/mol) for investigated complexes, along with energy contributions (kcal/mol). The values are given relatively to the investigated structure 1.

Complex	1	2
Nuclear repulsion energy	0	273,541.28
Electronic energy	0	−273,600.12
ΔE_nuc_ + ΔE_elec_	0	−58.84
Enthalpy	0	−57.87
Electronic entropy	0	0.00
Vibrational entropy	0	−2.62
Rotational entropy	0	−0.16
Translational entropy	0	0.00
Total entropy change	0	−2.78
Gibbs free enthalpy change	0	−55.09

## Data Availability

The original contributions presented in this study are included in the article. Further inquiries can be directed to the corresponding author.
